# Exploring Artificial Intelligence's Potential to Enhance Conventional Anticancer Drug Development

**DOI:** 10.1002/ddr.70182

**Published:** 2025-11-03

**Authors:** Sorin‐Ștefan Bobolea, Miruna‐Ioana Hinoveanu, Andreea Dimitriu, Miruna‐Andrada Brașoveanu, Cristian‐Nicolae Iliescu, Cristina‐Elena Dinu‐Pîrvu, Mihaela Violeta Ghica, Valentina Anuța, Lăcrămioara Popa, Răzvan Mihai Prisada

**Affiliations:** ^1^ Department of Physical and Colloidal Chemistry, Faculty of Pharmacy ”Carol Davila” University of Medicine and Pharmacy Bucharest Romania; ^2^ Innovative Therapeutic Structures Research and Development Centre (InnoTher) “Carol Davila” University of Medicine and Pharmacy Bucharest Romania

**Keywords:** artificial intelligence (AI), cancer, computational models, deep learning (DL), drug development, machine learning (ML), neural networks (NN)

## Abstract

Cancer affects one in three to four people globally, with over 20 million new cases and 10 million deaths annually, projected to rise to 35 million cases by 2050. Developing effective cancer treatments is crucial, but the drug discovery process is a highly complex and expensive endeavor, with success rates sitting well below 10% for oncologic therapies. More recently, there has been a growing interest in Artificial intelligence (AI) due to its potential to significantly enhance the success rates by processing large data sets, identifying patterns, and making autonomous decisions. The primary aim of this literature review is to examine the potential that state‐of‐the‐art AI tech‐nologies have to enhance and complement well‐established research methods used in cancer drug development, such as QSAR, interactions prediction, and ADMET prediction, among others. The basic technical aspects of computational technologies are clarified, and key terms commonly asso‐ciated with AI are defined. Current applications and case studies from academia and industry are presented to highlight AI's potential to accelerate progress in cancer drug research. Challenges and disadvantages of AI are also acknowledged, and it is discussed that future research should focus on overcoming its limitations to maximize its impact in cancer treatment.

## 1 Introduction

One in three to four people are at risk of developing cancer in their lifetime (Hayat et al. 2007; Sasieni et al. 2011; Zheng et al. 2023; Roy and Saikia 2016). Cancer is a group of genetic diseases characterized by uncontrollable growth and spread of abnormal cells in the body (Sarkar et al. 2013; Brown et al. 2023; Chaudhry et al. 2022; Vogt 1993). The International Agency for Research on Cancer (IARC) estimates that in 2022, around 20 million new cases of cancer have been discovered worldwide and close to 10 million deaths occur each year due to cancer (Bray et al. 2024; Zaimy et al. 2017; Sung et al. 2021). Moreover, the projected cancer burden is increasing: over 35 million new cancer cases are predicted in 2050—a 77% increase from the estimated 20 million cases in 2022 (Bray et al. 2024; World Health Organization 2024). These statistics reflect the fact that there is a pressing need to understand cancer and, most importantly, to transpose the current knowledge into effective and safe therapies. Therefore, innovation in healthcare and, more specifically, in cancer treatment discoveries, must be encouraged and supported.

In this light, cancer drug discovery and development are crucial in identifying new therapeutic targets, screening potential lead compounds, and assessing drug efficacy and safety (Singh et al. 2023; Wang, Song et al. 2023; Yang et al. 2022). It is widely recognized that the pipeline of drug discovery is extremely multifaceted and consists of numerous stages (Chan et al. 2019; Pandiyan and Wang 2022; Paul et al. 2021; Chen et al. 2023; Farghali et al. 2021). Before commencing the actual drug development process, it is essential to highlight the hypothesis and establish the target that will be studied subsequently, providing evidence regarding the assumed therapeutic effect of the candidate drug (Qureshi et al. 2023; Hughes et al. 2011). Following target validation, the next step is the identification and optimization of the lead molecule: this lead essentially represents a chemical compound active during primary and secondary assays, demonstrating adequate affinity, specificity, and selectivity for the target receptor. Consequently, the stages also include product characterization, formulation and development, preclinical research, and clinical trials, with all results and evidence being included in a New Drug Application that will be assessed by the competent authority for approval (Deore et al. 2019; Ator et al. 2006; Mohs and Greig 2017; Erakovic Haber and Spaventi 2017; Tamimi and Ellis 2009).

Nevertheless, after more than a decade of complex research with estimated 2.8 billion USD dedicated into the study and development of new drug entities, the failure rate remains very high across all therapeutic areas worldwide, with less than 10% of new entities reaching the market, majority of them failing the clinical phase (Pandiyan and Wang 2022; Qureshi et al. 2023; Vijayan et al. 2022; Gallego et al. 2021). Cancer drug development success rate sits well below the 10% average, with 97% of new cancer drugs failing the clinical trials phase, with an estimated of 1 in 20,000–30,000 drugs progressing from initial development to marketing approval (Gallego et al. 2021; Zhou et al. 2023).

The implementation of artificial intelligence (AI) in the drug development phase aims at improving these success rates, while increasing the accuracy and speed of the process (Shimizu and Nakayama 2020; Cui, Aouidate et al. 2020; Pasrija et al. 2022; Blanco‐González et al. 2023; Tripathi et al. 2021). The concept of AI was first introduced in a proposal for the Dartmouth Conference in 1956, authored by a collective of mathematicians and scientists, this event being credited with establishing AI as an independent field of study (Gallego et al. 2021; Chen et al. 2021; Bhinder et al. 2021). AI can be simply defined as the field of study that aims to create, test and optimize machines, systems or programs capable of performing tasks that would normally require human intelligence, such as learning, reasoning, problem‐solving, and decision‐making (Wang, Song et al. 2023; Pandiyan and Wang 2022; Paul et al. 2021; Chen et al. 2021; Bhinder et al. 2021; Zhang et al. 2023). From the 1950s to 2020 s, AI development has experienced periods of advancement and stagnation, the launching of ChatGPT chatbot by OpenAI on 30th of November 2022 seeming to spark an unprecedented interest in AI and its integration in numerous (if not all) domains of people's daily lives (Gallego et al. 2021; Blanco‐González et al. 2023; Jiménez‐Luna et al. 2021; Temsah et al. 2023; Terranova et al. 2024; Walters and Barzilay 2021).

Due to technical advancements in domains like statistics and computer science, computational methods like AI, machine learning (ML), and its subset, deep learning, have been successfully developed to assist in the discovery and development of drugs (Wang, Song et al. 2023; Zhang et al. 2023; You et al. 2022; Hasselgren and Oprea 2024). Computational methods present the advantage of being able to explore large volumes of data, along with the capability to distinguish and interpret patterns and the capacity to learn and issue independent decisions (Paul et al. 2021; Fan et al. 2021; Visan and Negut 2024; Qi et al. 2024; Moingeon 2021). Such algorithms can be employed at each step of the drug discovery and development process: target identification and validation, hit identification, hit to lead optimization, preclinical development, clinical development (Pandiyan and Wang 2022; Vijayan et al. 2022; Gallego et al. 2021; Visan and Negut 2024).

In recent years, many reviews have explored the topic of AI techniques for their transformative potential in drug discovery and development. Authors like Dhudum et al. (Dhudum et al. 2024), Abbas et al. (Abbas et al. 2024), and Bechelli and Delhommelle (Bechelli and Delhommelle 2024) outlined in their work the role of AI and ML in accelerating each stage of drug development, while providing a comparison with conventional drug discovery and development processes. Authors like Yang et al. (Yang et al. 2019). and Bhowmick et al (Bhowmick et al. 2025) also emphasize the future directions of computational methods in pharmaceutical research and development, while Vemula et al. (Vemula et al. 2023) and Sidiqqi et al. (Siddiqui et al. 2025). discussed the synergy of computer‐aided drug design (CADD) combined with AI, ML, and DL models in accelerating drug discovery.

The primary aim of this literature review is to examine how state‐of‐the‐art AI technologies can enhance and complement research methods that are already well‐established in cancer drug development (such as QSAR, interactions prediction, and ADMET prediction, among others). The basic technical aspects of computational technologies are clarified, and key terms commonly associated with AI are defined. Current applications and case studies from academia and industry are presented to highlight AI's potential to accelerate progress in cancer drug research. Examples of recently developed models used in drug research are offered. Furthermore, the advantages are considered alongside the existent limitations of AI technologies. It is discussed that future directions in research should investigate ways to overcome AI disadvantages and strengthen the technical capabilities, for the ultimate resolution to support and improve the cancer drug development process.

## 2 Search Strategy and Selection Criteria

The PubMed, Scopus and Google Scholar databases were searched for studies based on the search string: (AI OR ML OR deep learning OR deep‐learning OR natural language processing OR large language models) AND (cancer OR oncology OR antineoplastic OR anticancer OR anticancer OR chemotherapy OR immunotherapy OR radiotherapy OR radiation therapy OR cancer treatment) AND (drug development OR drug discovery OR drug design OR de novo drug design OR drug repurposing OR optimization OR drug‐target OR drug‐ligand OR target affinity OR druggability OR virtual screening OR ADMET OR ADME OR LADME OR LADMET OR QSAR OR CADD). Additional literature searches were performed in the Google Scholar databases, to examine details about AI techniques and models. The titles and the abstracts of the publications were screened based on their relevance to the topic, with the aim of determining the inclusion within our analysis. Full‐text review was performed in cases when the abstract was not sufficient to determine article inclusion.

Overall, upon selection and review of all literature references collected, the publications that have been assessed as relevant for the purpose of this report were studied and included within the present literature review.

## 3 Synthesis of Findings

### 3.1 AI

**Figure 1:**
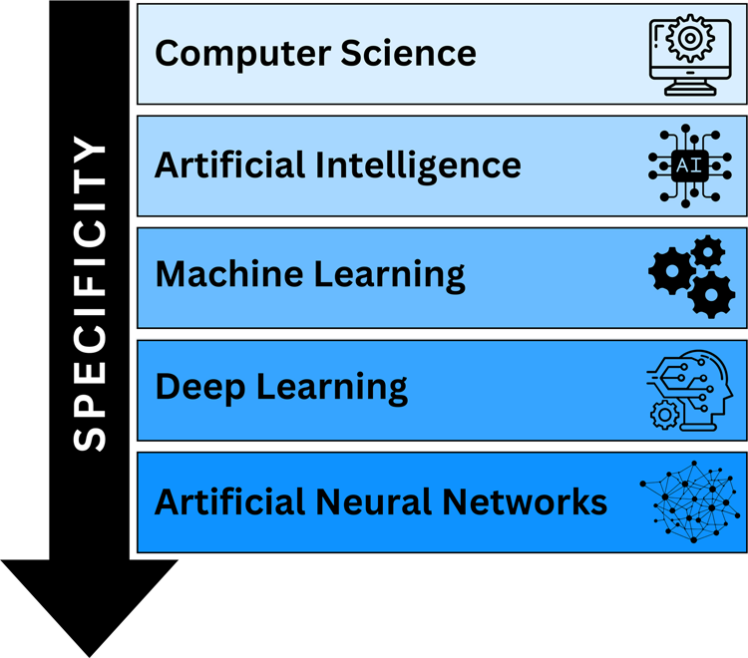
Hierarchy of common computational methods ranked on specificity (adapted from Choi et al. 2020).

AI is a term used to describe the field of science that aims at developing artificial entities like computers and robots that are able to emulate human intelligence in performing cognitive tasks like sensory perception, learning, reasoning, problem solving, and decision‐making (Amisha et al. 2019; Al Kuwaiti et al. 2023; Krishnan et al. 2023; Chang et al. 2019; Habehh and Gohel 2021; Hulsen 2023; Capobianco 2022). Artificial intelligence does not represent one single technology, but rather a broad set of technologies that rely on computer algorithms devised by humans or learned autonomously by the computational method to analyze large scales of data to support decisions or execute tasks (Figure 1) (Davenport and Kalakota 2019; Rowe 2019; Farina et al. 2022; Rubinger et al. 2023; Pettit et al. 2021). Artificial intelligence is commonly associated with “big data” and the ever‐increasing processing power of modern computers, but also incorporates elements of fields like languages, mathematics, and psychology (Mir et al. 2023; Hulsen 2022).

Artificial intelligence encompasses various techniques like ML, deep learning (DL), neural networks (NN), natural language processing (NLP), and large language models (LLMs) (Hulsen 2022, 2023; Alowais et al. 2023; Jiang et al. 2017). These methods will be further explained in detail.

ML is a subtype of artificial intelligence which involves creating computer models that are able to learn from experience and come up with algorithms that issue predictions and decisions without being explicitly programmed by humans (Al Kuwaiti et al. 2023; Rubinger et al. 2023; Grech et al. 2023; Hamilton et al. 2021; Kufel et al. 2023). ML models learn through iterations by applying mathematical and statistical models to training data sets with the aim of being able to make independent predictions about data points outside of the original training set (Shimizu and Nakayama 2020; Pettit et al. 2021; Hamilton et al. 2021; Sim et al. 2023). The difference from classical programming stems from the fact that classical programming uses data and algorithms to generate an output, while ML uses data and outputs to train machines so they can generate an algorithm (Pettit et al. 2021; Choi et al. 2020; Keskinbora and Güven 2020). ML is mainly categorized as “supervised” or “unsupervised” (Jiang et al. 2017; Bajwa et al. 2021). In supervised learning, a model is trained on labeled data, where each input is connected to an output (Habehh and Gohel 2021; Hamilton et al. 2021; Bajwa et al. 2021; Graham et al. 2019). This way, the model learns to associate inputs to outputs, enabling it to make predictions on new, unseen data, making it a suitable method for classification and regression (Noorbakhsh‐Sabet et al. 2019; Jovel and Greiner 2021). In unsupervised learning, the model is trained to extract information from unlabeled data with the aim to identify hidden patterns and create its own algorithms, being most often used in clustering, associations, and dimensionality reduction rather than prediction (Habehh and Gohel 2021; Hamilton et al. 2021; Bajwa et al. 2021; Graham et al. 2019; Sarker 2021). Supervised learning is implemented by ML algorithms like logistic regression, Support Vector Machine, decision tree, random forest, Naïve Bayes, k‐nearest neighbors, and artificial neural networks, while unsupervised learning is implemented by ML algorithms like k‐means algorithm, hierarchical clustering, deep belief networks, generative adversarial networks, and convolutional neural networks (Habehh and Gohel 2021; Uddin et al. 2019; Eckhardt et al. 2023; Raza et al. 2021). There are also subcategories like semi‐supervised learning which acts like a hybrid between unsupervised learning and supervised learning and reinforcement learning where the algorithm improves its decision‐making capabilities by trial and error through feedback from an interactive environment (Habehh and Gohel 2021; Hamilton et al. 2021; Graham et al. 2019; Noorbakhsh‐Sabet et al. 2019). ML models are increasingly being studied for their applications in cancer drug research, including drug discovery, drug repurposing, clinical trial design, efficacy and toxicity prediction, and drug combination optimization (Issa et al. 2021; Badwan et al. 2023; Wang et al. 2020).

Deep learning (DL) is a specific category within ML that is based on neural networks comprised of several nested layers of neurons modeled on the human brain which can handle more complex tasks (Chang et al. 2019; Pettit et al. 2021; Grech et al. 2023; Tran et al. 2021). Unlike ML, which is seen as a method of ‘statistical learning’ that uses a single layer network to generate output, deep learning is a more advanced method that operates with multiple layers of neural networks which enable the computer to train with much larger volumes of data and to improve itself with each iteration (Chang et al. 2019; Keskinbora and Güven 2020; Hashimoto et al. 2018). This allows the models to work with increased accuracy and specificity and less interpretability, such models showing improvements in performance compared to top‐performing ML algorithms (Shimizu and Nakayama 2020; Habehh and Gohel 2021). Deep learning models can automatically distinguish patterns from raw data, not needing humans to manually choose key features, unlike traditional ML models that require this step before training (Baptista et al. 2023; Parekh and Jacobs 2019; Alzubaidi et al. 2021). Like ML, deep learning methods can also be classified into supervised, unsupervised and semi‐supervised (Alzubaidi et al. 2021). Deep learning approaches are being increasingly used in medical science, including oncology and throughout all stages of drug development (Chen et al. 2024; Tan et al. 2020; Askr et al. 2023).

**Figure 2:**
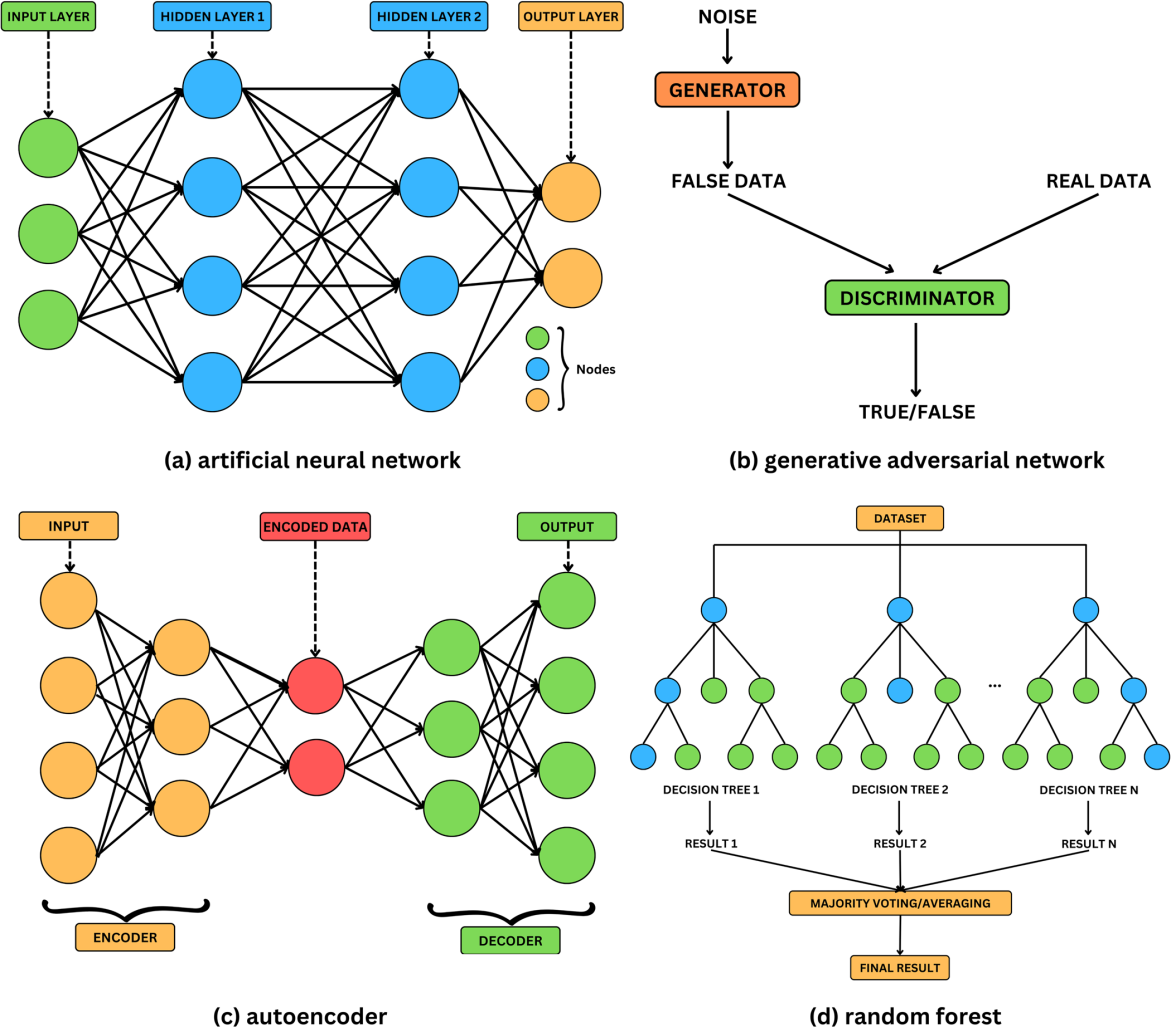
Graphical representation of (a) an artificial neural network, (b) a generative adversarial network, (c) an autoencoder, (d) random forest (adapted from Shimizu and Nakayama 2020).

An artificial neural network (ANN) is a computer model inspired by the biological structure and function of neurons. It comprises an input layer that receives data, one or more hidden layers that process the input through weighted connections by applying an activation function, and an output layer (Figure 2) (Shimizu and Nakayama 2020; Habehh and Gohel 2021; Davenport and Kalakota 2019; Hashimoto et al. 2018; Khan et al. 1998; Imai et al. 2020). Each layer consists of a set of nodes, also referred to as “neurons” or “units,” where each neuron in a layer is typically connected to neurons in the subsequent layer (Shimizu and Nakayama 2020; Habehh and Gohel 2021; Davenport and Kalakota 2019). A neuron receives input from multiple neurons and transmits signals to multiple neurons (Han et al. 2018). Each unit of the hidden layers processes the data from the input layer, taking into consideration the weight of the input signal and its intensity, and transfers it further along to succeeding units until it reaches the output layer (Shimizu and Nakayama 2020; Kufel et al. 2023; Imai et al. 2020; Dey 2022). The multilayer structure of the ANNs fosters data analysis efficiency, self‐organizing and self‐learning ability, strong information synthesis ability, rapid recognition of patterns, handling of noisy or incomplete data, and the capacity to solve complex problems, among others (Prisciandaro et al. 2023; Mao et al. 2020; Cao et al. 2021).

Natural language processing (NLP) is a subfield of AI that uses computer algorithms to automatically process human language with the aim to understand it, interpret it, and generate human language in its turn (Alowais et al. 2023; Crema et al. 2022; Yew et al. 2023). NLP achieves that by transforming written text into data sets that a statistical or ML model can process (Harrison and Sidey‐Gibbons 2021). NLP can be generally classified into natural language understanding and natural language generation (Khurana et al. 2023). NLP is used for tasks like text analysis, speech recognition, translation, information extraction, and other objectives related to language (Alowais et al. 2023; Graham et al. 2019; Undru et al. 2022). With regard to the medical field, some applications of NLP models include diagnostics automation, adverse events detection, decision support, and clinical outcome prediction (Buchlak et al. 2022).

Large language models (LLMs) are advanced natural language processing models able to perform tasks such as translations, text interpretation, processing and generation, as well as answering questions in a conversational manner with close resemblance to human capability (Cascella et al. 2024; Iannantuono et al. 2023; Clusmann et al. 2023). To perform such tasks, LLMs use neural networks and are trained on vast and diverse sets of text data, making them familiar with a large array of topics (Clusmann et al. 2023; Bitterman et al. 2024; Oniani et al. 2024). There are three predominant LLMs categories: encoder‐only LLMs with its representative Bidirectional Encoder Representations from Transformers (BERT), decoder‐only LLMs as exemplified by Generative Pre‐trained Trans‐former (GPT) models and encoder‐decoder LLMs represented by Text‐to‐Text Transfer Transformer (T5) and Bidirectional and AutoRegressive Transformers (BART) (Cascella et al. 2024). From the aforementioned, GPT models have the widest recognition, much due to the introduction to the public of ChatGPT in November 2022 by OpenAI, the chatbot achieving over 1 billion monthly users by March 2023 (Clusmann et al. 2023; Hershenhouse et al. 2024). Other LLMs include Google's Gemini and PaLM 2, Chatsonic, Perplexity, Anthropic's Claude, Meta's Llama 3, and GPT‐4 (Iannantuono et al. 2023; Clusmann et al. 2023). LLMs which are trained with text alongside other types of data are known as multimodal large language models (MLLMs) and can find applications in endeavors such as textual description of microscopic images (Cheng 2024; Truhn et al. 2024). Large language models are increasingly studied for their potential use in the field of medicine due to the fact that they can communicate with humans in plain language and their capability to use deduction and logic for applications such as data collection and information extraction, clinical documentation management, literature mining, aiding in diagnoses, therapeutic decision‐making, biomarker development, precision medicine, and drug development (Cascella et al. 2024; Bitterman et al. 2024; Truhn et al. 2024; Tayebi Arasteh et al. 2024; Meng et al. 2024; Tripathi et al. 2024).

### 3.2 Applications of AI in Cancer Drug Discovery and Development

In recent years, industry and academic researchers have recognized the immense potential of artificial intelligence to transform drug discovery and development. AI's ability to rapidly analyze vast amounts of data, identify patterns, and make predictions has opened new avenues for accelerating the discovery of novel therapies or improving existing ones. Consequently, models based on ML and its subset, deep learning, have been increasingly used within pharmaceutical research and development. Reports have shown that prominent industry names, including Takeda, Sanofi, Genentech, and Merck, have already implemented artificial intelligence in specific steps of the drug discovery process (Qi et al. 2024; Gupta et al. 2021).

**Figure 3:**
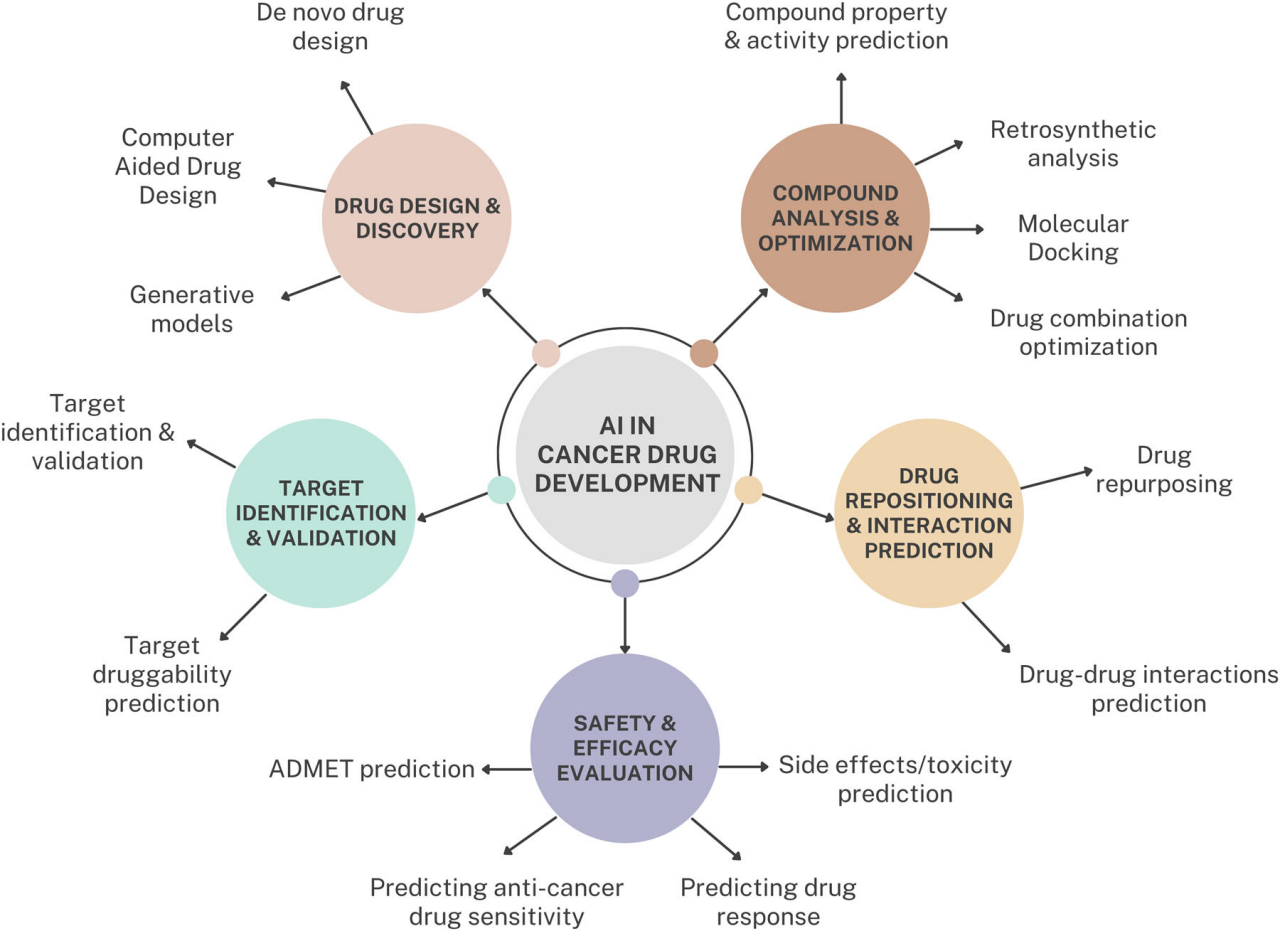
Applications of artificial intelligence in cancer drug discovery and development.

This subsection delves into how different cancer drug discovery and development methods benefit from artificial intelligence integration, showcasing how the computational models enhance and complement processes ranging from target identification and de novo synthesis to drug–drug interactions (DDIs) prediction and drug repurposing (Figure 3). Real‐life examples of implementation by researchers in academia or industry are also presented for each application, demonstrating the practical advantages and potential outcomes of AI‐driven approaches in advancing cancer therapeutics.

#### 3.2.1 Target Identification and Validation

Target identification is the process of finding the right drug targets for a disease while target validation confirms that affecting these targets impacts disease biomarkers and outcomes (Qureshi et al. 2023; Finan et al. 2017). Acknowledging that this specific process is pivotal in drug discovery, properties such as gene expression are fundamental for depicting genetic causes or mechanisms of action for specific diseases (Gupta et al. 2021; Pun et al. 2023). Nevertheless, the complexity of changes and alterations in the human genome when it comes to cancer presents a significant challenge for the process of identifying potential drug targets (Cortés‐Cros et al. 2013). Artificial intelligence assets have become popular solutions for the potential drawbacks of target identification and validation, with complex architectures that facilitate this pursuit and reduce the number of experiments and financial resources needed (Qi et al. 2024; Gupta et al. 2021; Najm et al. 2021; Kumari et al. 2015; Hu et al. 2019). These approaches make use of the increasing availability of large‐scale human genomics and proteomics data sets, being able to efficiently handle high throughput, heterogeneous, and complex molecular data (You et al. 2022; Dezső and Ceccarelli 2020).

The literature provides a multitude of examples of computational approaches employed for target identification and validation. For instance, in 2019, Madhukar et al. developed a ML model based on a Bayesian approach—BANDIT. Bayesian algorithms have been extensively used for various purposes, such as statistical analysis or optimization of candidate selection in de novo drug design (Ruberg et al. 2023; Colliandre and Muller 2024). The instrument encompasses multiple data types, providing an accuracy of around 90% for predicting the targets for more than 2000 small molecules. When applied to ONC201, a molecule under clinical development designated for the treatment of cancer, BANDIT identified and validated DRD2 as its target. Additionally, this model revealed dopamine receptor 2 as an unexpected but experimentally validated target for ONC201. The results of BANDIT in clinical research and development were monumental, generating approximately 4000 novel molecule‐target predictions (Madhukar et al. 2017, 2019).

In 2020, Zeng et al. designed deepDTnet, a deep learning model based on network analysis, aiming at in silico identification of drug targets. The enhanced precision of deepDTnet has facilitated approval from the US Food and Drug Administration for the identification of novel targets to pre‐existing drugs (Qi et al. 2024; Askr et al. 2023; Zeng et al. 2020; Dara et al. 2022). Integrating 15 different types of networks, from chemical to phenotypic and cellular, deepDTnet identifies relevant properties by learning representations of low‐dimensional vectors, for both drugs and their targets. Precisely, the algorithm is composed of two stages: first, a deep neural network architecture is used for network embedding, resulting in a low‐dimensional vector; thereafter, the prediction of drug‐target interaction is concluded using a PU‐matrix completion algorithm. The research was further conducted to experimentally validate the inhibitory activity of topotecan on human ROR‐γt and, moreover, to assess its therapeutic effects against multiple sclerosis in rodents (Askr et al. 2023; Zeng et al. 2020). In the same year, Dezső and Ceccarelli (Dezső and Ceccarelli 2020) performed a ML prediction of oncology drug targets based on protein and network properties, showing that the method is highly predictive on a validation data set consisting of 277 clinical trial drug targets, confirming that their computational approach is an efficient and cost‐effective tool for drug target discovery and prioritization. In 2022, Danishuddin et al. (Danishuddin et al. 2022) developed a LSTM (Long Short‐Term Memory) framework to predict lead molecule activities against seven cancer kinases, achieving accuracies of 0.81–0.78 and ROC‐AUC scores of 0.8–0.99, showing promise for new cancer drug development.

AlphaFold is an artificial intelligence system developed by DeepMind. By integrating numerous deep learning innovations, AlphaFold predicts the three‐dimensional structure of proteins from their amino acid sequences with accuracy comparable to experimental‐scale resolution, becoming an invaluable tool in structural biology (Keskin Karakoyun et al. 2023; Nussinov et al. 2022). There are a multitude of examples in literature on AlphaFold's uses in anticancer drug development. For example, in 2023, Keskin Karakoyun et al. (Keskin Karakoyun et al. 2023) presented the first structural analysis of 26 hereditary cancer genes, highlighting that AlphaFold‐derived confidence scores as a strong predictor of missense variant pathogenicity, outperforming traditional stability predictors in accuracy and robustness. In 2024, Wu et al. (Wu et al. 2024) demonstrated the utility of AlphaFold variants in accurately modeling TCR‐peptide‐MHC (T cell receptor‐peptide‐major histocompatibility complex) structures, providing insights into TCR recognition of NRAS neoantigens for immunotherapy. In the same year, Jones et al. (Jones, Miyauchi et al. 2024) used AlphaFold to reveal significant structural differences in the HPV E5 protein between low‐ and high‐risk genotypes, highlighting its potential role in HPV‐associated cancer development and Mikhaylov et al. (Mikhaylov et al. 2024) introduced an AlphaFold‐based pipeline for accurate modeling of peptide‐MHC complexes, outperforming existing tools and advancing structural immunology and neoantigen prediction.

#### 3.2.2 Target Druggability Prediction

Target druggability prediction refers to the evaluation of the measure in which a drug is capable to interact with and modulate a target (Pun et al. 2023; Zhou, Zhang et al. 2024; Vatansever et al. 2021). The human proteome contains thousands of potential pharmacological targets, but only a small fraction can be targeted by drugs (You et al. 2022). Therefore, efficiently pinpointing drug targets and evaluating their druggability are essential steps in discovering potential medications. However, traditional experimental methods, while accurate, are slow and not suited for high‐throughput screening. The drug discovery process also faces challenges such as limited resources, high costs, and a low hit‐to‐lead ratio. To address these issues, researchers are increasingly employing computational methods early in the workflow, providing a holistic view of molecules by integrating considerations of sequence, structure, and function. This approach fosters the development of more precise and cost‐effective strategies for identifying potential drug targets (Kandoi et al. 2015; Han et al. 2007; Bakheet and Doig 2009).

Advancements in computational modeling and algorithms have significantly enhanced drug‐disease network construction, allowing for a better understanding of drug effects on targets across various diseases, which complements traditional experimental techniques due to the high‐throughput nature of computational approaches (Bakheet and Doig 2009; Jamali et al. 2016; Lindsay 2005). ML‐based techniques offer a faster alternative for predicting druggable proteins, enabling rapid analysis of vast biological data sets and accurate assessment of anticancer targets druggability, while minimizing both time and costs associated with experimental validation (You et al. 2022; Serrano et al. 2024). These techniques are supported by algorithms like random forest, support vector machine, and convolutional neural networks (Vatansever et al. 2021).

Tools like SPIDER, CAVITY, and PockDrug make effective use of computational methods, the latter being employed by Yang et al. in 2021 to identify TNIK as a novel potential drug target in thyroid cancer (Charoenkwan et al. 2022; Yuan et al. 2013; Yang et al. 2021). In 2022, Raies et al (Raies et al. 2022) created DrugnomeAI, a ML framework predicting druggability likelihood for human exome genes. Integrating data from 15 sources, it generates predictions based on known drug targets, with emphasis on protein–protein interaction networks. The tool offers generic and specialized models by disease type or drug modality. Another tool for target druggability prediction is CancerOmicsNet, an AI model employing graph‐based algorithms, developed by Singha et al. (Singha et al. 2023) to predict cancer cell responses to kinase inhibitor treatments. Its performance was assessed through large‐scale benchmarking calculations and subsequent experimental validation across multiple cancer types. In 2024, López‐Cortés et al. (López‐Cortés et al. 2024) presented an innovative ML‐based method for predicting druggable proteins that drive cancer, which utilizes three sets of protein sequence descriptors: amino acid composition, di‐amino acid composition, and tri‐amino acid composition. The model was able to predict the druggability of 2339 cancer‐driving proteins, with 88.9% predicted to have druggable activity.

#### 3.2.3 Generative Models

Generating new molecules that can be used as anticancer drugs is a laborious task with high failure rates (Pereira et al. 2022). Computational generative models have successfully been developed to identify novel molecules leading to rising interest from academia and industry due to the potential of improved efficiency in de novo drug design (Joo et al. 2020; Wang et al. 2024; Martinelli 2022; Wang, Wang et al. 2022). Generative models are a form of neural networks that are able to learn and produce new data based on the existing data that it was trained on, including novel structures generated by merging parts of existing compounds (Qureshi et al. 2023; Badwan et al. 2023; Bordukova et al. 2024). The most representative generative model of deep learning is the generative adversarial network (GAN) (Kim et al. 2021). GANs can use chemical fingerprints, SMILES, molecular graphs, three‐dimensional structures, and other molecular representations for generating molecules with a desired property (Qureshi et al. 2023). Molecular fingerprints are vector representations that encode the characteristics of molecules. Depending on the type of molecular characteristics encoded, a distinction is made between structure key‐based fingerprints (based on molecular topology—e.g., the presence of benzene rings, carboxyl groups), path‐based fingerprints (enumerating the connectivity to specific substructures—e.g., a double bond to an oxygen atom), circular fingerprints (following also the structure, but describing holistically the surrounding environment of each atom in the molecule up to a predefined radius) (Baptista et al. 2022; Zagidullin et al. 2021).

In 2022, Pereira et al. (Pereira et al. 2022) applied a deep generative model for the generation of USP7 putative inhibitors; the framework managed to generate promising compounds, with more than 90% of them containing drug‐like properties and essential active groups for the interaction with the target. In the same year, Li et al. (Li et al. 2022) used generative deep learning that led to the discovery of a potent and selective RIPK1 inhibitor, while Wang et al. (Wang, Hsieh et al. 2022) proposed a new 3D‐based generative model called RELATION used to design inhibitors for two targets, AKT1 and CDK2, with favorable results. In 2023, Yu et al. (Yu et al. 2023) reported the discovery of a highly potent and selective macrocyclic CDK2 inhibitor via generative models and structure‐based drug design that showed robust antitumor efficacy in an OVCAR3 ovarian cancer xenograft model via oral administration.

#### 3.2.4 Property Prediction

Physicochemical properties like aqueous solubility, lipid solubility, partition coefficient, ionization decree, and intrinsic permeability are essential factors that impact the drug's interaction with the human body and influence the therapeutic answer and other phases of ADMET (Paul et al. 2021; Chen et al. 2023; Qureshi et al. 2023). Currently, most newly discovered drug candidates have undesirable physicochemical properties, such as poor solubility, low stability, poor distribution, or poor absorption (Bohr and Memarzadeh 2022). A lot of potential anticancer compounds fall into that category, making in silico property prediction an increasingly sought after method in the design and development phase of oncologic therapies (Terranova et al. 2024; Amara et al. 2023; Grebner et al. 2022; Miljković et al. 2021). These highly accurate computational methods employ ML and artificial intelligence algorithms to analyze large existing experimental and computational data to generate accurate predictions of compound physicochemical properties (Paul et al. 2021; Rodríguez‐Pérez and Bajorath 2021; Jia et al. 2022; Keshavarzi Arshadi et al. 2020). This approach allows for a faster exclusion of nonsuitable molecules with reduced time and other resources expenditure (Paul et al. 2021). Chemical and biological databases like the ones listed by Qureshi et al. (Qureshi et al. 2023). in 2023 are often used. Some AI tools used for property prediction were reviewed by Nosrati and Nosrati (Nosrati and Nosrati 2023) in the same year.

In 2017, Kumar et al. (Kumar et al. 2017) performed a prediction of human intestinal absorption of compounds using six AI techniques: k‐nearest neighbor, support vector machine, artificial neural network, partial least square, linear discriminant analysis, and probabilistic neural network, highlighting that support vector machine (SVM) with radial basis function based kernel may constitute a useful tool to estimate intestinal absorption at preliminary states of drug design and development. In 2021, Paul et al. (Paul et al. 2021) enumerated a list of use‐cases of artificial intelligence in compound properties prediction, including in quantitative structure–property relationship (QSPR), defining AI as a valuable tool in the prediction of physicochemical attributes of novel compounds, suggesting that this approach could be a better alternative to the time consuming trial‐and‐error process.

Aqueous solubility is an important physicochemical property in the discovery and development of anticancer drug candidates, as it significantly influences the compound's pharmacokinetics and formulation strategies (Cui, Lu et al. 2020). Artificial intelligence solubility prediction tools have been employed with success in this aspect. In 2020, Cui et al. (Cui, Lu et al. 2020) constructed deeper‐net models of ~20‐layer modified ResNet convolutional neural network architecture for predicting aqueous solubility which outperformed four established tools, shallow‐net models, and four human experts. In 2021, Meftahi et al. (Meftahi et al. 2021) conducted aqueous solubility prediction by QSPR modeling, obtaining a coefficient of regression (*R* ^2^) between 0.87 and 0.95 for models created using artificial neural networks. In 2023, Zhu et al. (Zhu et al. 2023) assessed 39 QSPR models based on 13 ML algorithms to predict aqueous solubility, the XGB model based on SRM showing the most promise.

#### 3.2.5 ML‐Enhanced Quantitative Structure–Activity Relationship (QSAR)

QSAR models are tools used to correlate the biological activity of substances with their chemical structure, as characterized by a selection of chemical descriptors (Ancuceanu et al. 2019; Singh et al. 2024; Karampuri and Perugu 2024). QSAR modeling streamlines chemical prioritization for desired biological activities through in silico methods, reducing the need for extensive in vivo or in vitro testing. Although conventional QSAR methods employing mathematical and statistical models have their merits, a transition is being made to artificial intelligence models, namely ML and its subset, deep learning (Singh et al. 2024; Karampuri and Perugu 2024). ML, including linear regression, support vector machines (SVM), Bayesian neural networks, and notably random forest (RF), enhances QSAR prediction, offering high predictability, simplicity, and robustness (Hessler and Baringhaus 2018; Zakharov et al. 2016; Fernandez et al. 2011). Also, unlike traditional drug discovery methods that depend on known chemical entities, AI‐enhanced QSAR can analyze large data sets of chemical compounds, including those that have not yet been synthesized or experimentally tested, using automated algorithms to uncover patterns and relationships between chemical structures and biological activity (Singh et al. 2024; Pusparini et al. 2023).

It is paramount to prioritize the development of precise and understandable QSAR models within the drug discovery continuum. Deep neural networks (DNNs), renowned for their potency as supervised learning tools, have demonstrated considerable potential in tackling regression and classification hurdles across diverse research domains, including pharmaceuticals. The principal proposed algorithms encompass deep belief networks (DBNs), convolutional neural networks (CNNs), dropout, autoencoder, Hessian free optimization (HF), rectified linear units (ReLUs) as a replacement for the sigmoid function, and conditional restricted Boltzmann machines (RBM) (Ghasemi et al. 2018; Xu 2022; Dahl et al. 2014).

Recent advancements have seen a surge in applications for predicting properties or activities, such as physicochemical and ADMET properties. These developments underscore the efficacy of this technology in facilitating quantitative structure–property relationships (QSPR) and quantitative structure–activity relationships (QSAR), all aimed at the discovery of enhanced anticancer agents (Speck‐Planche et al. 2012).

In 2023, Pusparini et al. (Pusparini et al. 2023) proposed a QSAR framework utilizing deep learning to predict estrogen receptor alpha inhibitors, establishing five classification models for predicting these inhibitors in breast cancer. Among these, the MATH model exhibited robust performance, suggesting its potential to assist in identifying candidate compounds for estrogen alpha inhibitors. In 2024, Gomatam et al. (Gomatam et al. 2024) presented a ML‐based QSAR approach for the informed prediction of PARP‐1 activity, an important target in the treatment of breast cancer, the generated results and insights having the potential to guide medicinal chemists in the design of novel inhibitors for this target. In the same year, Ding et al. (Ding et al. 2024) employed ML and Topomer CoMFA to build structural activity relationship models of potentially new VEGFR‐2 inhibitors, successfully identifying five promising anticancer compounds. Furthermore, Karampuri and Perugu (Karampuri and Perugu 2024) developed a breast cancer‐specific combinational QSAR model using 11 ML and deep learning techniques, 52 breast cancer cell lines, 25 anchor drugs, and 51 library drugs. Among the methods, deep neural networks (DNNs) showed particular promise, achieving an *R* ^2^ coefficient of 0.94.

Other recent examples from 2024 of AI‐enhanced QSAR in the field of anticancer drug development are highlighted in numerous publications. Ivanov et al. (Ivanov et al. 2024) was focused on developing QSAR models for TBK1 inhibitors using ML and molecular descriptors, with the goal of enhancing drug discovery for TBK1‐targeted therapies. Karampuri et al. (Karampuri et al. 2024) developed a multimodal deep neural network (MM‐DNN)‐based QSAR model to predict drug candidates targeting RTK signaling in breast cancer, achieving high predictive accuracy and identifying potential drug candidates. Duo et al. (Duo et al. 2024) used ML‐based virtual screening to identify novel compounds targeting SOS1 in the RAS signaling pathway, revealing promising inhibitors with unique binding modes and favorable drug‐like properties for RAS‐driven cancers. Park and Kang (El Rhabori et al. 2024) innovated upon traditional QSAR approaches by explicitly conveying differences between active and inactive compounds—thus not treating each structure independently as established through traditional QSAR models, but focusing on the structural features that differentiates the active from the inactive compound. This approach was made possible by popular ML algorithms, such as Support Vector Machine, Random Forest, XGBoost and Multi‐Layer Perceptron for model learning, with Support Vector Machine performing better than the others in terms of accuracy, area under curve, precision, and specificity. Mi et al. (Mi et al. 2024) which developed an AI‐QSAR model to predict the efficiency of nanoparticle tumor delivery and tissue biodistribution in tumor‐bearing mice after intravenous administration, using the physicochemical characteristics of nanoparticles and tumor therapy approaches.

#### 3.2.6 Predicting Ligand‐Receptor Interactions and Evaluating Binding Affinity

The initial phase in the drug discovery pipeline is to identify molecules that bind with strong affinity to the receptor site, which can be further transformed into drug‐like molecules (lead compounds) (Hughes et al. 2011; Dhakal et al. 2022). Identifying new drugs and their targets is still a challenging task due to the limited understanding of the dynamic relationship between chemical and genomic space (Dobson 2004; Kanehisa et al. 2006; Stockwell 2000). Experimental techniques for identifying lead compounds, such as high‐through put screening, can be costly and time‐consuming (Hughes et al. 2011; Dhakal et al. 2022). Conversely, computer prediction of protein‐ligand interaction (PLI) can eliminate the requirement for physical experimental research to screen for potential treatments, and also, can significantly reduce the resources, time, and expense involved. Hence, a trustworthy PLI predictive algorithm scan considerably accelerates the creation of novel therapies, eliminates toxic drug candidates, and effectively guides medicinal chemistry efforts (Dhakal et al. 2022; Kitchen et al. 2004).

ML algorithms take a different approach from classical virtual screening (VS) procedures (Sliwoski et al. 2014). In ligand‐based virtual screening (LBVS), it identifies new ligands by using information about the active ligand and the similarity between candidate ligands and known active compounds (Tresadern et al. 2009). Therefore, these methods demonstrate valuable advantages when 3‐dimensional structure of the target protein is unavailable. Furthermore, structure‐based virtual screening (SBVS) method screens compound libraries by using the three‐dimensional structure of a target (Lyne 2002). Conversely, ML employs the approach by utilizing the existing frameworks of protein‐ligand complex pairs to understand the relationship between physicochemical parameters and protein‐ligand interactions. This approach qualifies statistical models to forecast the status of other unspecified ligands/proteins.

Yamanishi et al. (Yamanishi et al. 2008) proposed in 2008 a kernel regression‐based method to infer protein–ligand interactions by combining the chemical structure information of ligands, sequence information of proteins, and the drug‐target complex network to find associations between drugs and target proteins, the results showing that some of the interactions identified by the method correspond to experimentally verified results in the literature. Similarly, another research conducted by Bleakley and Yamanishi (Bleakley and Yamanishi 2009) in 2009 utilizes a supervised learning method for investigating these interactions, demonstrating excellent performance in predicting four classes of drug‐target interaction networks involving ion channels, nuclear receptors, enzymes, and G protein‐coupled receptors in humans. In 2013, Wang and Zeng (Wang and Zeng 2013), within the framework of restricted Boltzmann machines, developed a technique to predict various types of interactions between proteins and ligands, going beyond binary contacts and including how they interact with each other. Their results indicate that the method is highly relevant for predicting drug–target interactions and for drug repurposing. In 2018, Öztürk et al. (Öztürk et al. 2019) introduced DeepDTA, a deep learning model designed to forecast the binding affinity between drugs and their respective targets. In DeepDTA, the drug and target were represented using SMILES (Simplified Molecular‐Input Line‐Entry System) and amino acid sequences, respectively, serving as input for a convolutional neural network (CNN). Building upon the foundation laid by DeepDTA, a succession of deep learning‐based models has been introduced, including WideDTA and DeepAffinity, which have emerged as valuable assets in terms of drug development (Öztürk et al. 2019; Karimi et al. 2019).

#### 3.2.7 Molecular Docking

Molecular docking is one of the most important and most often used methods in drug discovery and structure‐based drug design, being utilized in both industrial and academic settings (Sahu et al. 2024; Bortolato et al. 2013; Stanzione et al. 2021). Molecular docking is a computational technique which aims at predicting the interactions between a drug candidate which acts as a ligand and the binding site of a specific biological target (usually a protein) which acts like a receptor for the ligand (Agu et al. 2023; Ferreira et al. 2015; Trisciuzzi et al. 2018). In addition to the prediction of the binding mode between the molecules, this method also approximates the binding free energy of the ligand‐receptor complex (Bortolato et al. 2013; Ferreira et al. 2015; Chen et al. 2020; Sulimov et al. 2020). Docking is often used to perform virtual screenings of large libraries of compounds and ranking and choosing the best fitting molecule for a specific target (Morris and Lim‐Wilby 2008; Forli et al. 2016; Pagadala et al. 2017). Examples of such libraries are PubChem Compound Database, ZINC, Compound Database (AcD), and Cambridge Structural Database (CSD) (Fan et al. 2019). Since its initial development in the 1980s, molecular docking has evolved concomitantly with advancements on computer hardware and software, and today a multutitude of docking software and algorithms are available to use, some of the most representative being: Flex X, Gold, Glide, AutoDock, ZDOCK, RDOCK, LeDOCK, Dock, and AutoDock Vina (Stanzione et al. 2021; Ferreira et al. 2015; Fan et al. 2019).

Molecular dynamics (MD) simulations and free energy calculations have long served as conventional computational approaches to study molecular interactions (Deng and Roux 2009). MD simulations capture the time‐dependent behavior of biomolecules, revealing conformational flexibility and interaction stability, while free energy methods rigorously estimate binding affinities through thermodynamic calculations (Salo‐Ahen et al. 2021; Ahmed, Maldonado et al. 2023). Despite their accuracy, these methods are often computationally intensive and less scalable for screening large compound libraries (Grewal et al. 2025). In contrast, AI and ML techniques can rapidly analyze vast data sets, identify complex patterns, and complement classical methods by guiding simulations or refining predictions (Eastman et al. 2024; Galvelis et al. 2023).

In the case of oncologic drug discovery and development, molecular docking has been extensively deployed for the screening of potential drug candidates for various types of cancers. Poleboyina et al (Poleboyina et al. 2024). performed molecular docking to screen potential inhibitors of TGF‐β1 to prevent cervical cancer progression, nilotinib appearing to be a promising prospesctive; Yuan et al. (Yuan et al. 2021) investigated the mechanism of scopoletin against nonsmall cell lung cancer; Matada et al. (Purawarga Matada et al. 2022) investigated phytochemicals against EGFR, HER2, estrogen and NF‐KB receptors for their potential use in breast cancer; Meng et al. (Meng et al. 2020) used molecular docking to investigate 34 curcumin analogs as antiprostate cancer compounds; Alam et al. (Alam et al. 2021) used molecular docking among others to analyze and propose inhibitors against human liver cancer cell lines; Liñares‐Blanco et al. (Liñares‐Blanco et al. 2020) analyzed ademaciclib as a potential candidate for colon cancer; Sofi et al. (Sofi et al. 2022) investigated potential CDK1 inhibitors; Doan et al. (Doan et al. 2021) experimented with different glycyrrhetinic acid derivatives as anticolorectal cancer agents; Nagare et al. (Nagare et al. 2023) deployed molecular docking and simulation studies of flavanone and its derived compounds on pi3k‐akt pathway targeting against cancer; Yang et al. (Yang et al. 2023) used molecular docking to evaluate the mechanism of salidroside in the treatment of endometrial cancer; Sumera et al. (Sumera et al. 2022) used molecular docking and molecular dynamics studies reveal secretory proteins as novel targets of temozolomide in glioblastoma multiforme; Mahalakshmi Gunasekaran et al. (Gunasekaran 2022) used molecular docking analysis of lupeol with different cancer targets.

#### 3.2.8 ADMET Prediction

A fundamental challenge in pharmaceutical research is the prediction and optimization of pharmacokinetic properties, concerning absorption, distribution, metabolism, excretion, and toxicity (ADMET) which influence the efficacy and safety of a drug (Kim et al. 2020; Jia and Gao 2022; Tian et al. 2022). ML has been systematically employed to forecast ADMET properties of drugs using methods such as artificial neural networks, support vector machine regression, random forests, and Naïve Bayes, among others. Examples of computational tools supported by these methods include ADMETlab, ADMETlab 2.0, the Pharmacokinetics Knowledge Base (PKKB), the ADMET structure–activity relationship database (admetSAR), and admetSAR 2.0 (Visan and Negut 2024; Li et al. 2023).

Conventional approaches for evaluating ADMET properties often rely on experimental measurements and rule‐based computational models (Jamrozik et al. 2024). For example, partition coefficients (logP) are typically determined experimentally or estimated using fragment‐based or atom‐based computational methods to assess a compound's lipophilicity, which influences absorption and distribution (Klimoszek et al. 2024; Cevc 2015). Solubility is commonly measured in aqueous media under standardized conditions, providing insights into oral bioavailability (Savjani et al. 2012). These traditional methods serve as benchmarks for AI/ML predictions, allowing researchers to validate computational forecasts and integrate empirical data into predictive workflows for more accurate ADMET assessment (Jia and Gao 2022; Jamrozik et al. 2024).

In the case of predictive modeling, a combination of engineered and learned molecular descriptors is employed to achieve precise forecasts of human intestinal absorption. In vitro assessments, such as Caco‐2 cell permeability and parallel artificial membrane permeability, fulfill an essential role in predicting the potential for absorption. The development of predictive models concerning essential aspects such as drug‐protein binding, P‐gp inhibition, and blood‐brain barrier (BBB) permeability is facilitated by the availability of comprehensive data sets. Accurate predictions for various characteristics and interactions can be obtained by examining ML models, molecular descriptors, and structural patterns. These include BBB permeability, interactions with cytochrome P450 (CYP) enzymes as substrates or inhibitors, plasma half‐life, solubility, metabolic stability, potential metabolites, renal excretion, inhibition of bile salt export pump (BSEP), hepatotoxicity assessment, and cardiotoxicity (Singh et al. 2023; Dara et al. 2022; Tran et al. 2023; Sahu et al. 2022; Djoumbou‐Feunang et al. 2019; Kumar et al. 2021; Wu et al. 2020; Daina et al. 2017; Zhang et al. 2012; Maltarollo et al. 2015; Falcón‐Cano et al. 2022).

Previous articles have examined the influence of AI models on predicting various ADMET properties, such as CaCo‐2 permeability, carcinogenicity, blood–brain barrier permeability, and plasma protein binding (Wang, Song et al. 2023; Selvaraj et al. 2022). For example, in 2021, Vatansever et al (Vatansever et al. 2021). conducted an extensive review of state‐of‐the‐art methods in AI‐guided central nervous system drug discovery, with a particular emphasis on forecasting the blood–brain barrier permeability. In the same year, to predict the plasma protein binding of drugs, Mulpuru and Mishra (Mulpuru and Mishra 2021) developed a projection model that utilizes a chemical fingerprint and a freely available AutoML framework to determine the fraction of unbound drug in human plasma. In 2022, Selvaraj et al. (Selvaraj et al. 2022) examined the utilization of different ML models, such as SVM regression (support vector machine regression) and partial least squares (PLSs), for the prediction of the Caco‐2 permeability coefficient.

In 2022, Tian et al. (Tian et al. 2022) presented the web server ADMETboost comprised of an ensemble of features, including fingerprints and descriptors, and a tree‐based ML model (extreme gradient boosting) for ADMET properties prediction, the model performing well in the Therapeutics Data Commons ADMET benchmark group. In the same year, An et al. (An et al. 2023) developed a ML‐based approach to ERα bioactivity and drug ADMET prediction, the model demonstrating good results in forecast accuracy for those properties. In 2023, Li et al. (Li et al. 2023) used three ML algorithms (partial least squares‐discriminant analysis, adaptive boosting, and light gradient boosting machine) to predict ADMET properties of antibreast cancer compounds, the results showing that the LGBM model (light gradient boost machine) can be used as a reliable tool for researchers during the virtual screening of possible cancer drug candidates.

An industrial application of ADMET prediction based on computational models was presented by Fraczkiewicz et al. (Fraczkiewicz et al. 2015) in 2015, consisting of the collaboration between Simulations Plus and Bayer Pharma. Commercially available models developed by Simulations Plus include Gastro Plus and ADMET Predictor (Jones, Clark et al. 2024).

#### 3.2.9 Side Effects/Toxicity Prediction

With regard to the development of innovative medicine, the ability to anticipate side effects and evaluate toxicity during the preclinical phase remains of utmost importance, simultaneously mitigating the risk of later failure. Among the advantages of toxicity prediction, reducing the time and financial resources invested might be the primary objective to pursue in the preliminary phases of drug design (Tran et al. 2023; Zhang et al. 2018).

One of the most heavily invested prediction methods is QSAR, relying on parameters from chemical structures. QSAR‐based models have been used since the 1980s to predict toxicity, but have become more difficult to apply. Subsequently, the need for improvement became a top priority and artificial intelligence assets were considered viable solutions (Wu and Wang 2018; Dunn 1988).

Optimizing QSAR methods appeared to be the next logical step in advancing toxicity prediction, in silico approaches of ML being considered more valuable than in vivo and in vitro studies. The adoption of ML, due to its high accuracy and compensation for the limitations of previously used methods, has gained interest among researchers. Approaches that rely on computational predictions of toxicity have become extensively used for small molecules (Wu and Wang 2018; Galati et al. 2022; Uesawa 2020; Cavasotto and Scardino 2022).

For example, in 2017, Dimitri and Lió (Dimitri and Lió 2017) introduced DrugClust, a model constructed using advanced ML algorithms that aim to predict the side effects of various chemical compounds. Initially, the workflow involves categorizing the compounds based on similar properties, allowing a systematic approach to data analysis. Subsequently, the model leverages Bayesian scoring techniques to establish a framework for predicting potential side effects. In 2021, Li et al. (Li et al. 2021) developed DeepCarc, a model used to forecast the carcinogenicity of small molecules utilizing deep learning‐based model‐level representations. The model was developed using a data set of 692 compounds and evaluated on a test set of 171 compounds from the National Center for Toxicological Research liver cancer database (NCTRlcdb), showing good results and suggesting that DeepCarc can constitute an early detection tool for carcinogenicity assessment. In 2022, Galeano and Paccanaro (Galeano and Paccanaro 2022) developed a computational tool to forecast different scenarios, enabling improved management of toxicity risks. The approach included a matrix completion model, referred to as the Geometric Self‐Expressive Model (GSEM), which combines graphically structured information from chemical, pharmacological, and biological data sets.

VenomPred, a ML‐based platform, was designed by Galati et al. (Galati et al. 2022) in 2022 to predict the potential mutagenic, hepatotoxic, carcinogenic and estrogenic effects in small molecules. As a consequence of constant concern for development, in 2024, Di Stefano et al. (Di Stefano et al. 2024) developed VenomPred 2.0, an improved version of the platform, consisting of extensive endpoints that can undergo evaluation through an exhaustive consensus prediction strategy based on multiple ML models. Other similar computational model include DeepTox, developed by Mayr et al. (Mayr et al. 2016) in 2016 and ToxMPNN, created by Zhou et al (Zhou, Ning et al. 2024) in 2024, the former outperforming other models in capturing toxic features within the molecular structure.

#### 3.2.10 Drug Sensitivity

The ability to predict drug sensitivity plays a crucial role in the design and development of precision oncologic therapy that can in time lead to personalized treatment (Menden et al. 2013; Xu et al. 2023; Sharma and Rani 2020). The recent availability of large omics data sets like cancer cell lines led to the increasing interest for the development of ML approaches that are able to predict the response and the sensitivity of cancer cells to specific drugs (Sharifi‐Noghabi et al. 2021; Cortes‐Ciriano et al. 2017; Partin et al. 2021; Chawla et al. 2022). Deep learning, a rapidly progressing subset of ML algorithms, was employed in several studies to predict anticancer drug sensitivity (Tan et al. 2020; Baptista et al. 2021). These methods analyze vast cell‐line based data sets, such as National Cancer Institute 60, Cancer Therapeutics Response Portal (CTRP), Genomics of Drug Sensitivity in Cancer, Cancer Cell Line Encyclopedia, Genentech Cell Line Screening Initiative (gCSI) and estimate the drug response as a function of tumor characteristics and drug features (Xia et al. 2022).

The literature provides a multitude of use‐cases and proposed computational approaches to predict cancer drug sensitivity. In 2021, Gerdes et al. (Gerdes et al. 2021) presented Drug Ranking Using ML (DRUML), an approach which uses omics data to produce ordered lists of > 400 drugs based on their antiproliferative efficacy in cancer cells, the results indicating that DRUML accurately ranks anticancer drugs by their efficacy across a wide range of pathologies. In 2022, Chawla et al. (Chawla et al. 2022) introduced Precily, a predictive modeling approach to infer treatment response in cancers using gene expression data. The study's results suggest that sensitivity to anticancer therapy can be predicted in cancer cell lines with reasonable accuracy and reproducibility. In the same year, Shin et al. (Shin et al. 2022) proposed an interpretable model called DRPreter (drug response predictor and interpreter) that learns cell line and drug information with graph neural networks to be able to predict cancer cells sensitivity to oncologic drugs, the model outperforming other state‐of‐the‐art models in comparative experiments. In 2023, Liu and Mei (Liu and Mei 2023) proposed a novel drug sensitivity prediction (NDSP) model based on deep learning and similarity network fusion approaches, the method being able to extract highly interpretable biological features to achieve highly accurate sensitivity predictions of targeted and nonspecific cancer drugs. In 2024, Pang et al. (Pang et al. 2024) presented DrugGene, an interpretable DL model that integrates gene expression, gene mutation, gene copy number variation of cancer cells, and chemical characteristics of anticancer drugs to predict sensitivity, the model demonstrating higher accuracy than existing prediction methods, while being able to learn the reaction mechanisms between cancer drugs and cell lines from various features, and to interpret the model's predicted results.

#### 3.2.11 Drug Combination Optimization

In the case of cancer treatment, drug combinations have the potential to exhibit enhanced therapeutic efficacy with less unwanted outcomes like toxicity, side effects, and drug resistance (Fan et al. 2021; Rani et al. 2023). Due to the high order number of possible drug combinations, screening them unassisted is practically impossible (Zhou et al. 2023; Wang et al. 2020; Rani et al. 2023). Thus, AI methods are becoming more and more sought‐after tools when wanting to screen and optimize a drug combination. These methods include ML systems (artificial neural networks, support vector machine, random forest, logistic regression, stochastic gradient boosting, Bayesian models, network‐based modeling) and DL systems (deep convolution neural networks, recurrent neural networks, deep belief networks, deep Boltzmann machine, deep autoencoder learning), DL being the popular choice for modeling drug combination effects (Baptista et al. 2023; Tsigelny 2018; Vidyasagar 2015).

The literature provides a multitude of examples of implementation of computational approaches for drug combination optimization. For example, Pivetta et al (Pivetta et al. 2013). employed artificial neural networks with standard back‐propagation to predict the synergism of anticancer drugs. Experimental assessments of the drugs' cytotoxicity were conducted both individually and in combination on cell lines. Subsequently, the ANN was trained using the results of these experiments, utilizing 60 combinations with 15 allocated for validation. This system facilitated the evaluation of cytotoxicity for all potential combinations within the selected concentration range. In 2018, Preuer et al. (Preuer et al. 2018) introduced DeepSynergy, a deep learning method designed to forecast the synergy of drug combinations, accompanied by a comprehensive method comparison. DeepSynergy leverages both compound and genomic data as input. Through the inclusion of genomic data, DeepSynergy can discern between various cancer cell lines and identify specific drug combinations that exhibit maximum efficacy on a particular cell line. DeepSynergy integrates information about the cancer cell line and the drug combination within its hidden layers to construct a unified representation, ultimately resulting in precise predictions of drug synergies. In 2022, Preto et al. (Preto et al. 2022) presented SYNPRED, a system comprised of ensembles of AI algorithms, as well as links omics and biophysical traits that can predict drug combination effects in cancer. The model exhibited high performance characteristics when given an independent test set. In 2024, Abd El‐Hafeez et al. (Abd El‐Hafeez et al. 2024) presented a ML framework that finds synergistic combinations for FDA‐approved cancer drugs, the analysis highlighting that gemcitabine, MK‐8776 and AZD1775 as frequently synergizing across cancer types.

#### 3.2.12 Drug Repurposing

Over time, cancer cells can develop resistance to chemotherapy and the side effects after repeated administration can become unacceptable for the patient (Turabi et al. 2022; Pillai U et al. 2023; Xia et al. 2024). The discovery of new oncologic drugs is a costly and time‐consuming endeavor with high failure rates at all stages of the process (Issa et al. 2021; Ahmed, Kang et al. 2023; Dash et al. 2023). A possible alternative to avoid/mitigate the time and money expenditures associated with developing new therapeutic anticancer products is drug repurposing (Issa et al. 2021; Xia et al. 2024; Zhang et al. 2021; Weth et al. 2024). Drug repurposing is the process through which an existing drug, a shelved drug, or a drug that failed in the clinical phase is screened to be used for another indication than the ones initially intended when first developed (Xia et al. 2024; Yin and Wong 2021; K and Cho 2022; Khachigian 2020; Amiri et al. 2023).

Artificial intelligence techniques like ML have been systematically employed to further accelerate and improve the drug repurposing process (Issa et al. 2021; Tanoli, Vähä‐Koskela et al. 2021). This was made possible due to advancements in the fields of informatics, genomics and biology, and the general availability of large compounds, proteins, and diseases libraries (Bhattarai et al. 2016; Pan et al. 2022). This computational method approach implies the analysis of large‐scale input from such databases, including pharmacological, genetic, chemical, or clinical data, with the objective of pattern recognition and identification of connections between already approved drugs and new potential diseases they could treat (Visan and Negut 2024; Han et al. 2023; Zhao and So 2019; Siddiqui et al. 2022).

Two examples of databases used for in silico drug repurposing are Drug Repurposing Hub and RepurposeDB (Tanoli, Vähä‐Koskela et al. 2021). In 2021, Tanoli et al. performed a comprehensive survey on 102 databases supporting drug repurposing, highlighting how these databases can be employed for this endeavor, while providing examples of methods for predicting drug activity by using data from public databases (Tanoli, Seemab et al. 2021).

In 2021, Cui et al. (Cui et al. 2021) proposed GraphRepur—a graph neural network model based on GraphSAGE for drug repurposing against breast cancer. The model integrated two classes of computational methods, drug signature‐based and drug network‐based, outperforming previous state‐of‐the‐art approaches. In 2022, Wu et al. (Wu et al. 2022) developed DRviaSPCN: a software package for drug repurposing in cancer via a subpathway crosstalk network. DRviaSPCN was demonstrated to be an efficient tool for the identification of potential cancer drugs, showing promise as an asset in the drug development pipeline. In the same year, Shao et al. (Shao et al. 2022) performed a computational drug repurposing based on a recommendation system and drug‐drug functional pathway similarity. The model returned an accurate drug response prediction, while also providing an interpretable mechanism on drug effects at the same time.

#### 3.2.13 DDIs Prediction

DDIs happen when two or more medications are taken together (Cheng and Zhao 2014). Developing methods that can identify DDIs before drug approval and even before the clinical trial phase can significantly decrease patient morbidity and mortality due to medication errors (Cheng and Zhao 2014; Zhang et al. 2024; Mei and Zhang 2021). Numerous ML‐based techniques have been developed to forecast DDIs. There are generally two scenarios in DDI prediction methods: one aims to predict the presence of an interaction between two drugs, while the other focuses to identify the particular type of interaction, event, or effect that occurs between the drugs. The former situation can be treated as a binary classification problem, whereas the latter presents a multiclassification complexity (Zhang et al. 2024).

In the process of pharmaceutical development, the prediction of cytochrome P450 (CYP450) enzyme induction or inhibition is crucial because of the possible risks associated with DDIs. In 2019, Wu et al. conducted a study that employed a combination of ensemble learning and deep learning methods to accurately classify CYP450 inhibitors. With an accuracy rate of 90.4%, ensemble techniques, including random forest, gradient boosting decision tree, and eXtreme gradient boosting, demonstrated superior results compared to deep learning techniques. The SHapley Additive exPlanations (SHAP) method was utilized to explain the models and identify prospective DDIs during early drug discovery (Singh et al. 2023; Wu et al. 2019).

In 2022, Zhu et al. proposed a comprehensive multi‐attribute discriminative representation learning (MADRL) model to forecast DDIs. MADRL employs a generative adversarial network (GAN) to capture detailed information about DDI attributes, enabling accurate prediction of DDIs. The performance of the MADRL algorithm was assessed using an openly accessible data set (Wang, Song et al. 2023). In the same year, Chen et al. proposed 3DGT‐DDI, a unique approach that combines a pretrained textual attention module and a 3D graph neural network to increase prediction accuracy and deliver biological insights. The ability to predict DDIs was improved by the integration of location data and a 3D molecular graph structure, which was notably unique to 3DGT‐DDI. Experimental findings demonstrated that 3DGT‐DDI outperformed other existing models in terms of prediction performance (Wang, Song et al. 2023; He et al. 2022).

While numerous existing methodologies concentrate on forecasting the occurrence of interactions between two drugs, it is essential to also delve into the underlying mechanisms of DDIs. Consequently, Zhang et al. presented in 2020 a deep learning framework called DDIMDL that investigated the hidden mechanisms of DDI events and predicted the varieties of events using a diversity of drug attributes, achieving an accuracy of 0.8852 and an area under the precision‐recall curve of 0.9208 (Wang, Song et al. 2023; Deng et al. 2020).

#### 3.2.14 Retrosynthetic Analysis

Retrosynthetic analysis is a method through which the process of obtaining a drug molecule is reverse‐engineered from the target compound to simpler, commercially available materials together with the synthetic path from the raw materials to the final product (Bai et al. 2020; Wang, Pang et al. 2023; Lin et al. 2022; Yan et al. 2022). Computer aided synthesis planning can trace its root back to Corey's LHASA method in the 1960s, but recent advances in computer science and informatics have generated a great leap forward alongside an increasing interest in this tool for developing chemical compounds, especially in the field of medicine and pharmacy (Struble et al. 2020). Moreover, artificial intelligence, coupled with large data availability, has shown great potential for improving the computational methods for synthesis planning and retrosynthetic analysis (Baylon et al. 2019; Nair et al. 2019; Hasic and Ishida 2021). The methods employ ML algorithms and large chemical reactions data sets like Reaxys and the USPTO database in the search for possible retrosynthetic routes, while also scoring candidate pathways based on their potential to lead to the target product (Merzbacher and Oyarzún 2023; Gao et al. 2018; Watson et al. 2019). Examples of such used algorithms are Monte Carlo Tree Search (MCTS) and the A* search algorithm (Han et al. 2023; Zhao et al. 2024). Other examples of retrosynthesis models are NeuralSym, Seq. 2Seq, Retrosim, GLN, and MEGAN, all of which can return potential reactants for a specific given target compound (Zhong et al. 2023).

In 2021, Chen and Jung (Chen and Jung 2021) performed multistep retrosynthesis predictions by LocalRetro for lenalidomide, a cancer drug that can be used to treat multiple myeloma, results showing the exact same synthesis pathway as the one present in literature. In 2022, Urbina et al. (Urbina et al. 2022) used a retrosynthetic analysis software called MegaSyn on de novo generated lapatinib analogs (an orally active drug for breast cancer and other tumors) after testing the software on various approved anticancer drugs. In 2023, Faris et al. (Faris et al. 2023). conducted retrosynthesis via Synthetic Pathway Assembler (SPAYA) program on potential JAK3/STAT inhibitors, highlighting that significant time and resources can be saved by utilizing SPAYA during the synthesis of complex compounds.

### 3.3 Challenges and Disadvantages

Although AI‐driven cancer drug discovery stands out through its computing efficiency and high in silico success rates, there are limitations in terms of data input for system training, model interpretability, and validation protocols (Ching et al. 2018; Muratov et al. 2020).

First, large data sets are required to train neural networks, the model's ability to make predictions being highly dependent on data size, quality, and diversity (Ching et al. 2018; Watson et al. 2023). At the same time however, the field of drug development faces a lack of adequate experimental data and publicly available data repositories (Rifaioglu et al. 2019).

Another open challenge is model interpretability—that is, the way end users interpret the AI models to make testable hypotheses about the system under study (Askr et al. 2023; Ching et al. 2018; Watson et al. 2023). This is triggered by what was earlier pointed out to be the “black box” nature of ML models (meaning, the internal algorithms and understanding how and why the prediction is formed may not be readily approachable by the end users) (Askr et al. 2023; Gupta et al. 2021; Keith et al. 2021).

Additionally, the validation procedure (typically the second stage of the AI‐based drug discovery process, that follows data collection) could substantially improve model quality (Muratov et al. 2020; Pandey et al. 2022). Real data (e.g., high‐quality clinical data sets) are needed to validate the in silico therapy response forecasts (Askr et al. 2023).

**Figure 4:**
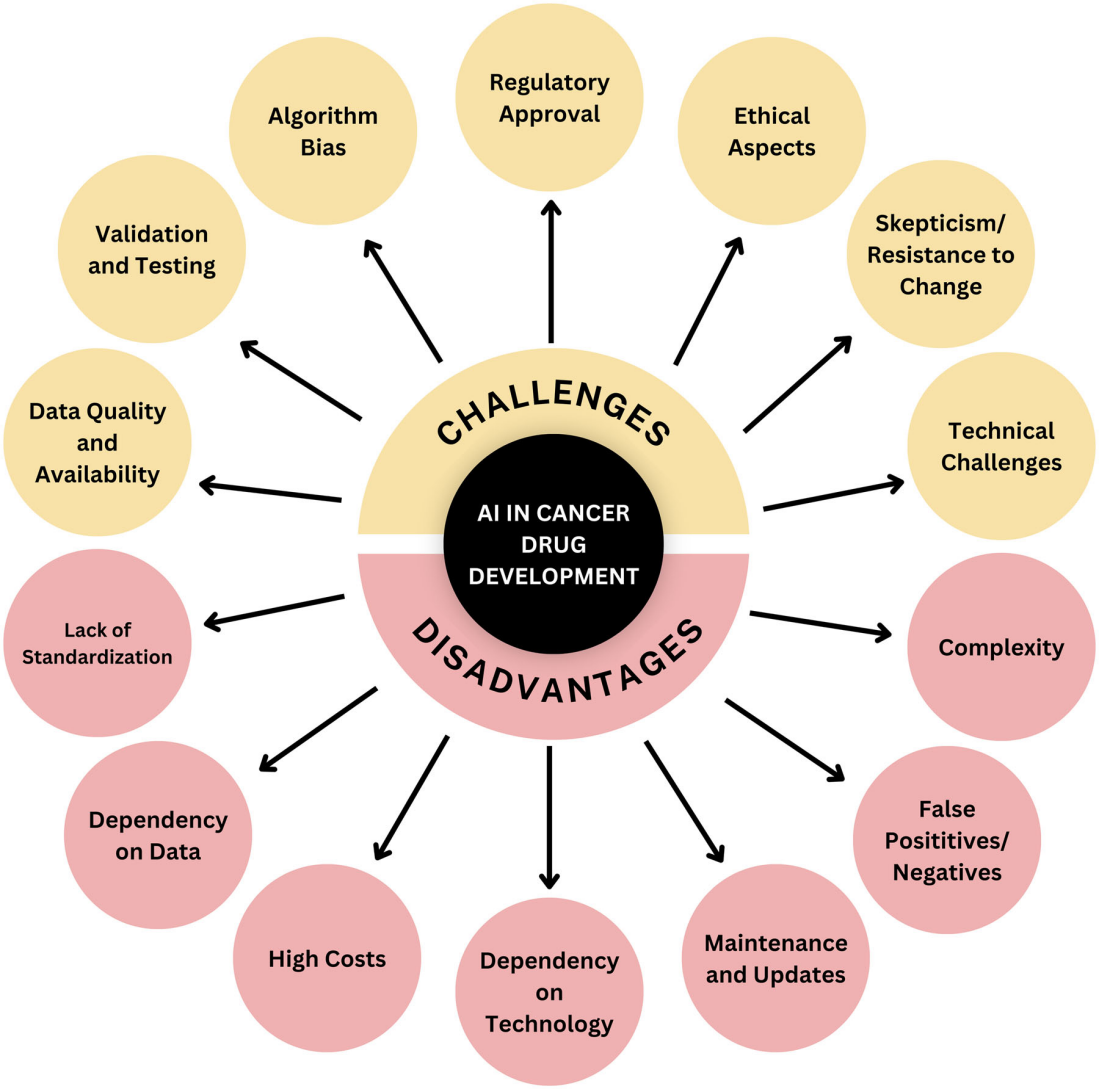
Challenges and disadvantages of implementing AI technologies into cancer drug development.

Challenges and disadvantages of implementation of artificial intelligence technologies into cancer drug development are graphically presented in Figure 4.

## 4 Conclusions and Future Perspectives

Technology entrepreneur and investor Naval Ravikant famously said that “competing without software is like competing without electricity.” Maybe that quote will 1 day stand true for artificial intelligence as well. Artificial intelligence is deemed to have the potential to lead a fourth industrial revolution, businesses and individuals seeming to work to integrate it into their day‐to‐day activities maybe much like internet or even electricity were adopted in their respective incipient eras. The evolution in technology during this era has considerably contributed to the development of AI.

Initially, the innovative AI approaches were limited to nonmedical applications, their utilization for improving healthcare outcomes now becoming progressively more prevalent worldwide. One area of healthcare that can use all edge it can get is the fight to develop safe and efficient therapies for cancer.

This paper presented how artificial intelligence and other computational methods can help advance the discovery and development processes for oncologic drugs. A brief introduction to the history of AI was presented, followed by descriptions of technologies situated under the umbrella of artificial intelligence. Then, branches of the drug development process that benefit from AI technologies were explored, from target identification to drug repurposing, providing real life examples of such models being implemented by academia or industry. Finally, we presented disadvantages of AI implementation in the oncologic drug discovery and development pipeline, alongside the challenges encountered in the process.

To address the challenges identified in the current bibliographic review, we propose that future research in AI‐driven cancer drug discovery should focus on creating curated gold‐standard data sets available to the community, developing benchmarks for comparing different interpretability approaches, and seeking consensus to evaluate the accuracy and reproducibility of discovery methods.

## Ethics statement

The authors have nothing to report.

## Consent

The authors have nothing to report.

## Conflicts of Interest

The authors declare no conflicts of interest.

## Data Availability

No new data were created or analyzed in this study. Data sharing is not applicable to this article.
